# Mutational Escape in HIV-1 CTL Epitopes Leads to Increased Binding to Inhibitory Myelomonocytic MHC Class I Receptors

**DOI:** 10.1371/journal.pone.0015084

**Published:** 2010-12-08

**Authors:** Yue Yang, Jinghe Huang, Ildiko Toth, Mathias Lichterfeld, Xu G. Yu

**Affiliations:** 1 Ragon Institute of Massachusetts General Hospital, Harvard and MIT, Boston, Massachusetts, United States of America; 2 Infectious Disease Division, Massachusetts General Hospital, Boston, Massachusetts, United States of America; Singapore Immunology Network, A*STAR, Singapore

## Abstract

Escape mutations in HIV-1 cytotoxic T cell (CTL) epitopes can abrogate recognition by the TCR of HIV-1-specific CD8+ T cells, but may also change interactions with alternative MHC class I receptors. Here, we show that mutational escape in three HLA-A11-, B8- and B7- restricted immunodominant HIV-1 CTL epitopes consistently enhances binding of the respective peptide/MHC class I complex to Immunoglobulin-like transcript 4 (ILT4), an inhibitory myelomonocytic MHC class I receptor expressed on monocytes and dendritic cells. In contrast, mutational escape in an alternative immunodominant HLA-B57-restricted CTL epitope did not affect ILT4-mediated recognition by myelomonocytic cells. This suggests that in addition to abrogating recognition by HIV-1-specific CD8 T cells, mutational escape in some, but not all CTL epitopes may mediate important immunoregulatory effects by increasing binding properties to ILT4, and augmenting ILT4-mediated inhibitory effects of professional antigen-presenting cells.

## Introduction

Due to its high genetic plasticity, HIV-1 can rapidly form escape mutations that confer resistance to antiretroviral drugs or abrogate recognition by adaptive immune responses[1]. This has been particularly clearly documented in the context of immune pressure mediated by HIV-1-specific CD8+ T cells, which can induce escape mutations in targeted epitopes that reduce peptide binding to the restricting HLA class I allele[2], interfere with recognition by TCR contact residues[3] or inhibit intracellular epitopic peptide processing[4]. However, in addition to abrogating recognition by HIV-1-specific CD8+ T cells, escape mutations in cytotoxic T cell (CTL) epitopes may also affect interactions of CTL epitope/MHC class I complexes with alternative MHC class I receptors. For instance, a common mutation in the immunodominant HLA-B*2705 restricted gag epitope KK10 (KRWIILGLNK) significantly enhances binding to ILT4, an inhibitory MHC class I receptor expressed on monocytes and dendritic cells and causes substantial defects in the antigen-presenting properties of such cells[5]. By altering interactions between peptide/MHC class I complexes and immunomodulatory MHC class I receptors on myelomonocytic cells, CTL escape mutations may therefore have important, previously unrecognized regulatory effects on the functional profile of dendritic cells and monocytes.

To test whether HIV-1 CTL escape mutations are commonly associated with altered recognition by myelomonocytic MHC class I receptors on myelomonocytic cells, we here focused on analyzing how escape variants in four immunodominant HIV-1 CTL epitopes change interactions between peptide/MHC class I complexes and the inhibitory myelomonocytic MHC class I receptor ILT4. The following epitopes and their variants were included: The HLA-B*0801 restricted nef epitope FLKEKGGL (FL8 wt) and its variants FLREKGGL (FL8 K3R) and FLKEEGGL (FL8 K5E), the HLA-A*1101 restricted gag epitope ACQGVGGPGHK (AK11 wt) and its frequent variant ACQGVGGPSHK (AK11 G9S), the HLA-B*0702-restricted gp120 epitope IPRRIRQGL (IL9 wt) and its common variant IPTRIRQGL (IL9 R3T) and the HLA-B*5701 restricted gag epitope TSTLQEQIGW (TW10 wt) and its variant TSTLQEQIAW (TW10 G9A). These naturally-occurring variants were all shown to result from CD8+ T cell mediated immune pressure in prior studies[2], [3], [6], [7].

## Results and Discussion

**Figure 1:**
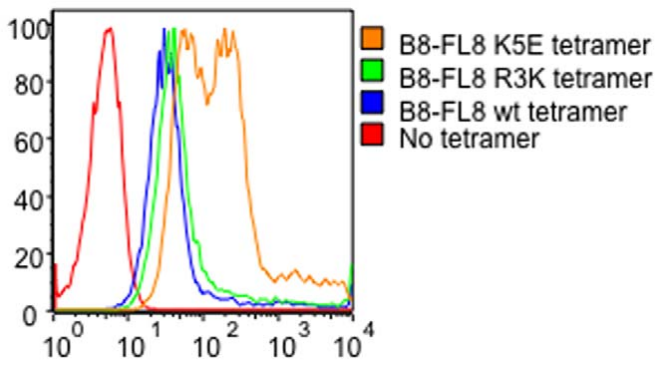
Representative flow cytometry dot plot reflecting binding of HLA-B8-FL8 wild-type and variant tetramers to CD14+ monocytes.

To analyze how HIV-1 CTL escape mutations alter binding properties of peptide/MHC class I complexes to MHC class I receptors expressed on myelomonocytic cells, we initially focused on the immunodominant B8-FL8 epitope. We observed that a major naturally occurring escape variant with an amino acid substitution at position 5 (K5E) in the epitopic peptide substantially increased binding of the respective tetramer to CD14+ monocytes as compared to the tetramer refolded with the wild type tetramer (Figure 1). An increase in tetramer/pentamer binding relative to the wild type tetramer/pentamer was also observed after staining of monocytes with a tetramer incorporating the K3R variant within the epitopic peptide, however, differences were less pronounced (Figure 1). We subsequently found that the two CTL escape variants in the A11-AK11 epitope (G9S) and the B7-IL9 epitope (R3T) also mediated stronger binding interactions of the corresponding peptide/MHC class I complexes to monocytes compared to the respective wild type epitopes. In contrast, no differences were observed between binding of all of these tetramers to HLA class I-mismatched T cells; moreover, CD8 T cells specific for the respective wild type epitopes were not stained by the corresponding variant tetramers (Figures S1, S2 and S3), indicating that the specific tetramer/pentamer binding patterns to monocytes were unrelated to the intrinsic fluorescence emission characteristics of these reagents. The differential tetramer/pentamer binding properties to monocytes were significantly reduced by antibodies that selectively blocked ILT4, while no effect of ILT4 antibodies on binding interactions between tetramers and HIV-1-specific CD8 T cells were observed (Figure S1, S2, S3). This demonstrates that the observed binding of tetramers to myelomonocytic cells was mediated by the inhibitory MHC class I receptors ILT4. However, blockade of ILT4 by antibodies did not fully abrogate binding of the respective tetramers/pentamers to monocytes, suggesting that interactions with alternative MHC class I receptors on myelomonocytic cells might contribute to the recognition of these tetramers/pentamers. Notably, monocytes express a series of alternative stimulatory and inhibitory MHC class I receptors that might be involved in interactions with HLA class I molecules[8]. Moreover, the ILT4-specific antibody used for this study might have a partial overlap with ILT2 and we therefore cannot exclude that some of the tetramer binding was mediated by recognition of ILT2. Yet, tetramer binding to primary monocytes or dendritic cells has never been shown to be influenced by ILT2 antibodies in our own [5], [9] and other previous studies [10].

**Figure 2:**
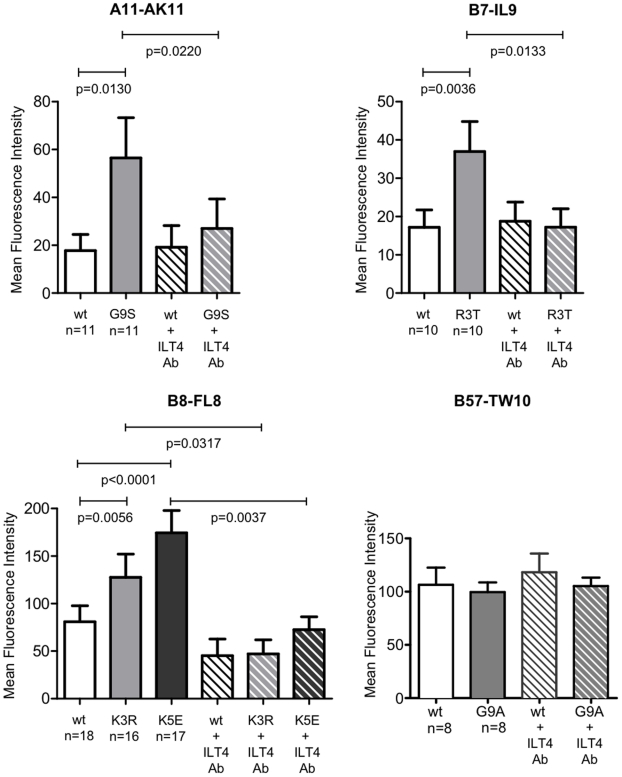
HIV-1 CTL escape mutations increase recognition of peptide/MHC class I complexes by the inhibitory myelomonocytic MHC class I receptor ILT4 on monocytes. Data represent the mean fluorescent intensity of MHC class I tetramers/pentamers refolded with the indicated epitopic peptide or peptide variant. Tests were performed in the indicated number of individuals. Data show mean and standard deviation. Significance was tested using paired t-test.

To test if increased recognition of HIV-1 CTL escape variants is a universal phenomenon, we additionally analyzed how a typical escape mutation at position 9 in the immunodominant B57-TW10 epitope influences recognition of the corresponding peptide/MHC class I complex by ILT4 on myelomonocytic cells. These experiments demonstrated no impact of this escape mutation in the epitope on interactions with ILT4 on monocytes (Figure 2). Overall, this suggests that a selected number of mutational escape mutations increase binding properties of the respective peptide/MHC class I complexes to ILT4, while other variants do not.

In this study, we show that in three randomly selected HIV-1 CTL epitopes, sequence variations induced by CD8+ T cell mediated immune pressure can lead to increased binding to the inhibitory myelomonocytic receptor ILT4 expressed on dendritic cells and monocytes. As shown in multiple prior studies, 2–3 fold higher binding of MHC class I complexes to ILT4 translates into a functional inhibition of the antigen-presenting and cytokine secretion properties of myelomonocytic cells[5], [9]; therefore it is reasonable to assume that the CTL variants studied here induce inhibitory functional effects on such professional antigen-presenting cells. Overall, these results are in line with the recent understanding that ILT4 can recognize MHC class I complexes[5] or CD1d molecules[11] in an antigen-specific fashion. Moreover, our data suggest that inhibitory, ILT4-mediated immunoregulatory effects of CTL epitope escape variants may represent a relatively common and previously underestimated biological consequence of HIV-1 sequence diversification. These inhibitory effects may contribute to the progressive decline of antigen-presenting properties in myelomonocytic cells during advanced HIV-1 infection that has been documented in a variety of prior investigations[12], [13]. Thus, our data emphasize that mutational escape in HIV-1 CTL epitopes may be associated with a complex pattern of immunoregulatory events that are mediated by altered recognition of CTL escape variants by myelomonocytic MHC class I receptors. Recent studies have proposed to include CTL epitope variants into sequences for HIV-1 vaccine candidates, which may facilitate the induction of HIV-1-specific CD8+ T cells with a broader ability to cross-recognize mutated variants[14]. However, inclusion of such variants may be associated with unwanted ILT4-mediated inhibitory immunoregulatory effects that decrease the functional properties of myelomonocytic cells and in this way may contribute to a lower immunogenicity of the immunogens. Thus, the immunoregulatory effects of CTL escape variants should be considered when selecting sequences for HIV-1 vaccine candidates.

## Methods

To investigate altered recognition of these escape variants by myelomonocytic MHC class I receptors, we used Phycoerythrin (PE)-labeled recombinant MHC class I tetramers or pentamers refolded with the respective wild-type or variant peptides. The B8-FL8 tetramers were produced at the NIH Tetramer Core Facility; the A11-AK11 tetramers were purchased from Becton Dickinson (San Diego, CA) and the B7-IL9 tetramers were obtained from ProImmune (Oxford, UK). Tetramers/Pentamers refolded with wild type and corresponding variant peptides were labeled with identical fluorophores, and were simultaneously synthesized at their respective production facilities. We tested recognition of these tetramers to circulating CD14+ monocytes from untreated chronically HIV-1 infected persons, who had been recruited at the Massachusetts General Hospital after written informed consent was obtained according to the Institutional Review Board-approved study protocol. Briefly, 2 ul of each tetramer/pentamer was incubated with 1×10^6^ freshly isolated PBMC for 20 minutes at room temperature; after 20 minutes, monoclonal CD14-specific antibodies were added. Cells were then incubated for additional 10 minutes, washed twice and processed to flow cytometric acquisition on an LSRII cytometer. To determine whether the observed binding of tetramers/pentamers to monocytes were mediated by interactions with the inhibitory myelomonocytic MHC class I receptor ILT4, experiments were conducted with PBMC that had been pre-incubated with polyclonal ILT4-blocking antibodies (R&D Systems) for 30 min at 37C prior to staining with MHC class I tetramers/pentamers.

## Supporting Information

Figure S1 **Binding of B7-IL9 wild-type tetramer and the corresponding variant tetramer (IL9 R3T) to HIV-1-specific CD8 T cells.** Dot plots indicate binding of the wild type and the respective variant tetramer to wild-type-specific CD8 T cells in the presence or absence of ILT4 blocking antibodies.
(TIF)

Figure S2 **Binding of A11-AK11 wild-type tetramer and the corresponding variant tetramer (AK11 G9S) to HIV-1-specific CD8 T cells.** Dot plots indicate binding of the wild type and the respective variant tetramer to wild-type-specific CD8 T cells in the presence or absence of ILT4 blocking antibodies.
(TIF)

Figure S3 **Binding of B8-FL8 wild-type tetramer and the corresponding variant tetramers (FL8 K5E and K3R) to HIV-1-specific CD8 T cells.** Dot plots indicate binding of the wild type and the respective variant tetramer to wild-type-specific CD8 T cells in the presence or absence of ILT4 blocking antibodies.
(TIF)
