# Supplementary figures and images for: Mutational Escape in HIV-1 CTL Epitopes Leads to Increased Binding to Inhibitory Myelomonocytic MHC Class I Receptors
Source: PLoS One. 2010 Dec 8;5(12):e15084. doi: 10.1371/journal.pone.0015084 (PMC2999561; doi:10.1371/journal.pone.0015084)

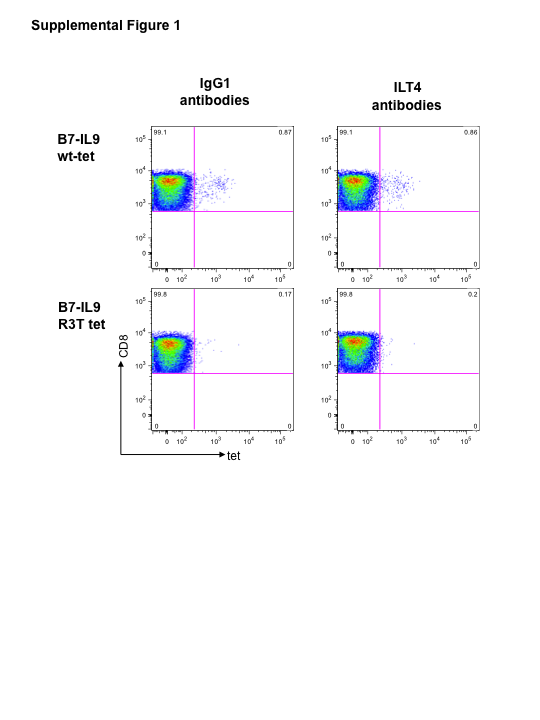

Supplement: Figure S1 — Binding of B7-IL9 wild-type tetramer and the corresponding variant tetramer (IL9 R3T) to HIV-1-specific CD8 T cells. Dot plots indicate binding of the wild type and the respective variant tetramer to wild-type-specific CD8 T cells in the presence or absence of ILT4 blocking antibodies. (TIF) [file pone.0015084.s001.tif]

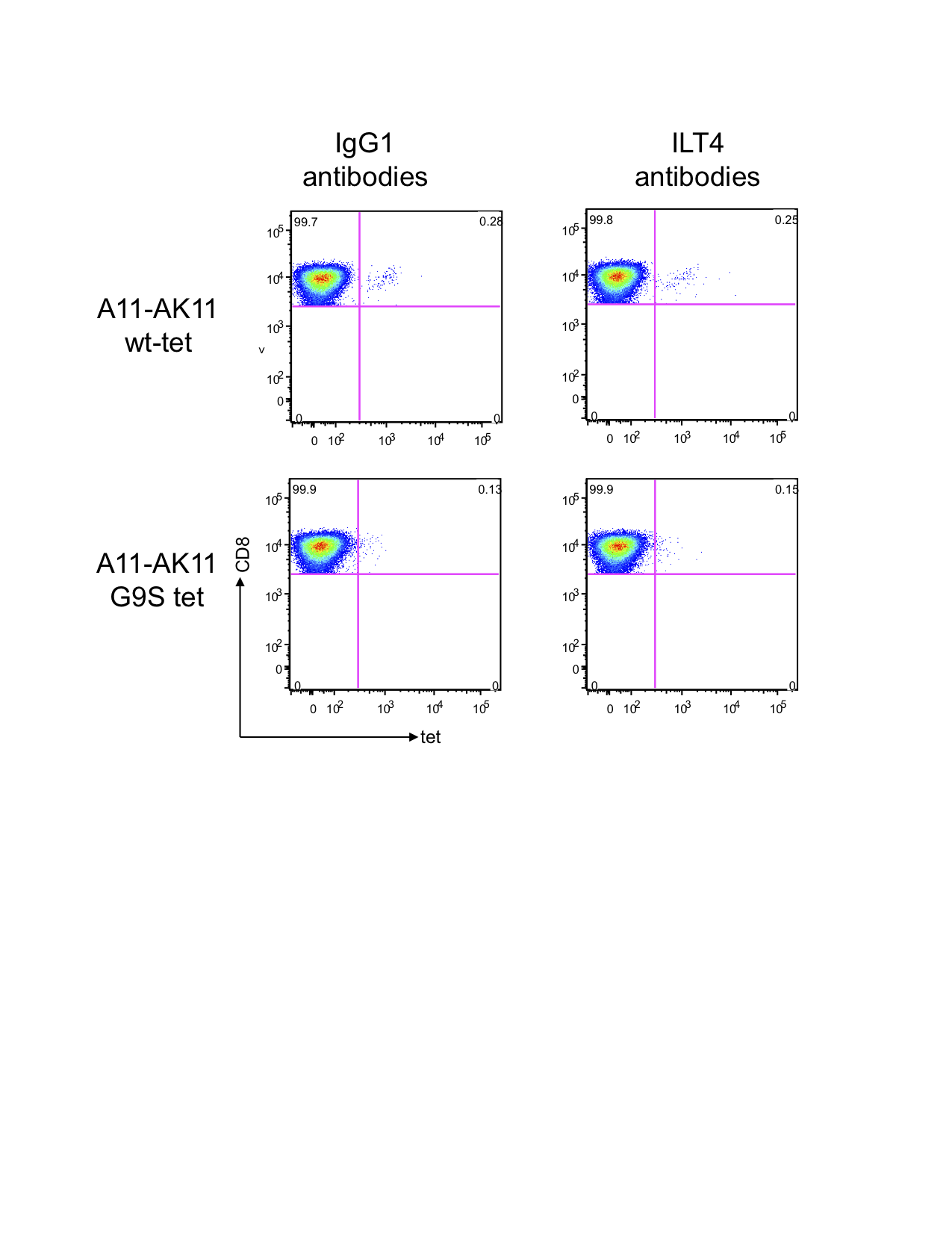

Supplement: Figure S2 — Binding of A11-AK11 wild-type tetramer and the corresponding variant tetramer (AK11 G9S) to HIV-1-specific CD8 T cells. Dot plots indicate binding of the wild type and the respective variant tetramer to wild-type-specific CD8 T cells in the presence or absence of ILT4 blocking antibodies. (TIF) [file pone.0015084.s002.tif]

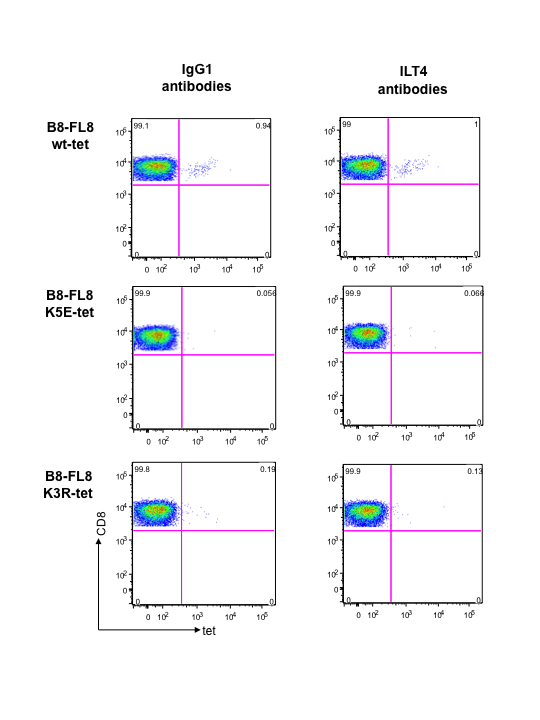

Supplement: Figure S3 — Binding of B8-FL8 wild-type tetramer and the corresponding variant tetramers (FL8 K5E and K3R) to HIV-1-specific CD8 T cells. Dot plots indicate binding of the wild type and the respective variant tetramer to wild-type-specific CD8 T cells in the presence or absence of ILT4 blocking antibodies. (TIF) [file pone.0015084.s003.tif]
